# In-silico Analysis of *NF1* Missense Variants in ClinVar: Translating Variant Predictions into Variant Interpretation and Classification

**DOI:** 10.3390/ijms21030721

**Published:** 2020-01-22

**Authors:** Matteo Accetturo, Nicola Bartolomeo, Alessandro Stella

**Affiliations:** 1 R&D Department, BioTechnology Services srl, 71122 Foggia, Italy; matteoaccetturo@yahoo.it; 2 Sezione di Igiene, Dipartimento di Scienze Biomediche e Oncologia Umana, Università degli Studi di Bari Aldo Moro, 70124 Bari, Italy; nicola.bartolomeo@uniba.it; 3 Laboratorio di Genetica Medica, Dipartimento di Scienze Biomediche e Oncologia Umana, Università degli Studi di Bari Aldo Moro, 70124 Bari, Italy

**Keywords:** variant interpretation, missense variants, NF1, VEST3, REVEL, ClinPred

## Abstract

*Background*: With the advent of next-generation sequencing in genetic testing, predicting the pathogenicity of missense variants represents a major challenge potentially leading to misdiagnoses in the clinical setting. In neurofibromatosis type 1 (NF1), where clinical criteria for diagnosis may not be fully present until late infancy, correct assessment of variant pathogenicity is fundamental for appropriate patients’ management. *Methods*: Here, we analyzed three different computational methods, VEST3, REVEL and ClinPred, and after extracting predictions scores for 1585 *NF1* missense variants listed in ClinVar, evaluated their performances and the score distribution throughout the neurofibromin protein. *Results*: For all the three methods, no significant differences were present between the scores of “likely benign”, “benign”, and “likely pathogenic”, “pathogenic” variants that were consequently collapsed into a single category. The cutoff values for pathogenicity were significantly different for the three methods and among benign and pathogenic variants for all methods. After training five different models with a subset of benign and pathogenic variants, we could reclassify variants in three sharply separated categories. *Conclusions*: The recently developed metapredictors, which integrate information from multiple components, after gene-specific fine-tuning, could represent useful tools for variant interpretation, particularly in genetic diseases where a clinical diagnosis can be difficult.

## 1. Introduction

In the past few years, the availability of high throughput sequencing technologies (Next Generation Sequencing, NGS) has dramatically increased the number of variants identified in disease-causing genes (DCGs). The majority of these variants are deposited in locus specific databases, such as ClinVar, an NIH-funded database, where observed variants and clinical annotations are reported. While for several DCGs pathogenicity could be the consequence of either loss-of-function or gain-of-function mutations, some other genes can be functionally impaired by more subtle mechanisms in combination with additive effects. In fact, both hypermorphic and hypomorphic alleles were recently reported in patients with dominant axonal Charcot-Marie-Tooth disease [1], and FMF-associated *MEFV* mutations were experimentally demonstrated to be of hypermorphic nature [2].

Therefore, although for many DCGs ClinVar hosts prevalently missense variants causing amino acid changes in proteins, for a large subset of these missense variants correct classification and interpretation remain elusive [3,4]. In addition, misclassification of variants can represent a serious challenge in gene testing results interpretation, due to several factors, such as clinical misdiagnosis, wrong assumptions about pathogenicity mechanisms, outdated entries [5].

To reduce uncertainty in variants classification, the American College of Medical Genetics (ACMG) and the Association for Molecular Pathology (AMP) jointly released interpretation guidelines that have been thoroughly assessed and refined in recent years [6,7,8,9,10,11]. The ACMG/AMP guidelines are based on the combination of multiple lines of evidence with variable rank translating a qualitative evaluation into a five-tier classification system. These guidelines have been a fundamental step in establishing a common set of interpretation rules. However, they did not eliminate discordancy across molecular genetics laboratories [7,12,13]. 

To further decrease ambiguity and to develop consensus methods for specific DCGs different working groups and expert panels tried to provide more specific criteria at single gene-disease level [14,15,16]. 

Tackling the still unresolved issue of accurate variant classification, has been the goal of a growing number of *in-silico* computational methods based on evolutionary conservation, protein structure and/or sequence homology. The first generation of these algorithms was usually focusing on specific features, as the likelihood that a specific amino acid change would affect protein function [17,18,19,20,21], or measuring the degree of conservation at specific amino acid positions [22,23,24]. A different set of computational methods has been specifically developed to assess the putative consequences of nucleotide changes on splicing proficiency [25,26,27,28]. A more recent, second generation of prediction tools combine and integrate information deriving from multiple methods evaluating different features as possible mechanisms leading to pathogenesis [29,30,31]. These in silico tools have been generally referred to as “ensemble methods” or “metapredictors”. However, both first- and second-generation computational methods have been trained on rather large sets of human genomic variants. Hence, their performances and predictions reliability may vary when applied to specific disease-causing genes. The utility of gene-specific tailoring using *in-silico* prediction tools is becoming progressively recognized [32], and has been considered useful to help in reducing the number of variants of uncertain significance (VUS). However, VUS still represents the large majority of variants reported in several human variants databases, including ClinVar [33].

Neurofibromatosis type 1 (NF1), caused by pathogenic variants in *NF1*, is an autosomal dominant disease which causes mainly cutaneous clinical manifestations, including cafè-au-lait (CALs) macules, axillary and/or inguinal freckling, neurofibromas, Lisch nodules of the iris, and subcutaneous or plexiform neurofibromas. A clinical diagnosis of NF1 is suspected when the minimum internationally recognized criteria set by NIH are present [34]. The *NF1* gene encodes for a protein called neurofibromin, and since its cloning [35,36], little progress has been made on its function. Neurofibromin, a huge 2818 amino acid (327 kDa) protein, is expressed ubiquitously although a higher level of expression is present in the adult peripheral and central nervous system [36]. In the central part of the protein is present, a 360 amino acids region named NF1-GRD (GAP-related-domain) homologous to the catalytic domain of the GTPase activating protein (GAP). This domain confers neurofibromin the ability to down-regulate the RAS pathway, thus, acting as a negative regulator of signals for cell proliferation and differentiation. For this reason, *NF1* is considered a tumor-suppressor. The oncosuppressor activity of *NF1* has been later confirmed by the findings of somatic *NF1* mutations in many human cancers, including breast cancer [37], ovarian cancer [38], lung cancer [39], glioblastoma [40] and acute myeloid leukemia [41]. This role has been reinforced by recent large pan-cancer genomic sequencing studies which revealed the presence of *NF1* alterations in 6% of patients’ tumors. Further, *NF1* was mutated in as many as 54 cancer types with the highest frequency occurring in different types of melanomas [42]. 

However, inferring the putative pathogenic role of variants causing neurofibromatosis type 1, which is associated with an increased risk of some peculiar cancer types, but mainly oculo-cutaneous features [43], is a challenging task. In fact, in 50% of cases, disease-causing mutations arise *de-novo* making co-segregation studies (an important asset in variant interpretations) not possible. In addition, NF1 clinical diagnosis is usually suspected early in childhood, while internationally recognized diagnostic criteria are not fully met until teenager years [44,45]. Thus, when non-obvious disease-causing mutations are identified, especially in children, genetic counsellors face gene testing results whose interpretations may remain uncertain at best, or until patient fully develop symptoms. 

Here, we aimed at assessing the performance of three recently developed metapredictors, VEST3, REVEL and ClinPred, applied to *NF1* gene missense variants reported at ClinVar. Indeed, of the 4464 *NF1* variants listed at ClinVar, nearly half have an uncertain significance classification, with most of them being missense variants.

We performed a comprehensive analysis of the *NF1* missense variants listed at ClinVar to verify the ability of these three in silico tools in correctly predicting variants’ pathogenic consequences, and we propose a more straightforward classification. We opted to select these three predictors, since they have been demonstrated to outperform, singularly or in combination, most currently available prediction algorithms [30,31,32,33]. Our findings may assist clinicians and diagnostic laboratories in selecting one or more computational tools as an aid to correctly interpret the pathogenicity of missense variants. Further, our approach may have a wider application in developing specific interpretation guidelines for other disease-causing genes.

## 2. Results

### 2.1. VEST3, REVEL and ClinPred Prediction Score Distribution for NF1 Missense Variants

First, we extracted the VEST3, REVEL and ClinPred scores for 1585 *NF1* missense variants (49 BENIGN/LIKELY BENIGN, 1364 VUS, 50 CI, and 122 LIKELY PATHOGENIC/PATHOGENIC) deposited at ClinVar. Next, we verified whether VEST3, REVEL and ClinPred scores for the classes “likely benign” vs. “benign”, and “likely pathogenic” vs. “likely pathogenic/pathogenic” vs. “pathogenic” were significantly different. 

In no cases, the medians of the two benign classes, and of the three pathogenic classification groups demonstrated statistically significant differences (Mann-Whitney test not significant for “benign” vs. “likely benign” comparison, Kruskal-Wallis test not significant for non-parametric ANOVA of “likely pathogenic” vs. “likely pathogenic/pathogenic” vs. “pathogenic”). Therefore, in the remaining statistical analyses, the *NF1* missense variants classified at ClinVar as “benign” and “likely benign” were merged into the category “LEANING BENIGN”. Similarly, the “likely pathogenic”, “likely pathogenic/pathogenic”, and “pathogenic” classes, were combined into the “LEANING PATHOGENIC” category. Variants classified as VUS and with conflicting interpretation (CI) maintained their respective ClinVar classification.

Since for all four categories “LEANING BENIGN”, “VUS”, “CI” and “LEANING PATHOGENIC” and for all predictors, the score distributions were again not normal (Shapiro-Wilk test, Supplementary Table S1), we proceeded to assess medians and 95% confidence intervals. 

**Figure 1:**
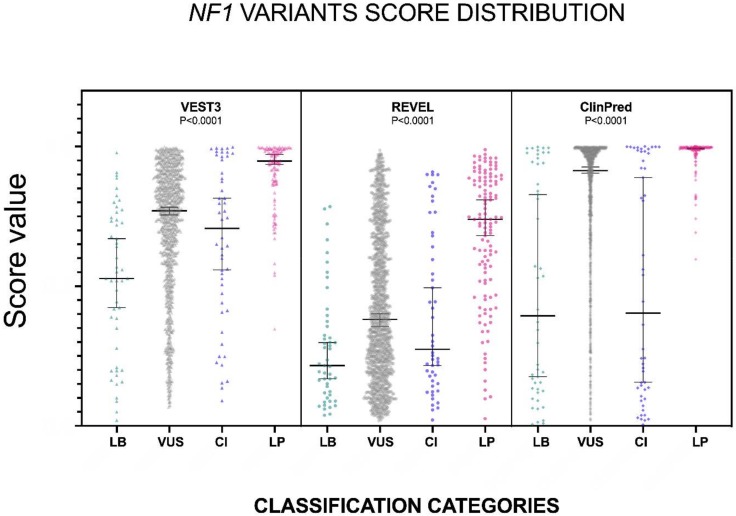
Prediction scores of the three computational methods for the LB (LEANING BENIGN), VUS (variants of uncertain significance), CI (conflicting interpretations), and LP (LEANING PATHOGENIC) *NF1* missense variants. Median and 95% confidence intervals are reported for each category. The P value of non-parametric ANOVA is reported for each predictor (Kruskal-Wallis test).

The distribution of the VEST3, REVEL and ClinPred scores, medians, 95% confidence intervals are reported in Figure 1 and in Table 1.

The non-parametric one way ANOVA of prediction scores distribution among categories was highly significant for all three metapredictors (*p* < 0.0001 for all predictors with VEST3 Kruskal-Wallis statistic K = 156.1, REVEL Kruskal-Wallis statistic K = 113, ClinPred Kruskal-Wallis statistic K = 168). The greatest difference between medians was among categories LEANING BENIGN vs. LEANING PATHOGENIC for all three metapredictors, and differences were highly significant in all cases. The widest variation was present between the LEANING BENIGN and LEANING PATHOGENIC categories for ClinPred (−0.599, *p* = 0.0004), while the smallest margin was observed between VUS and CI for both VEST3 and REVEL (0.063 and 0.107, respectively). In contrast, the difference between medians of ClinPred scores for the VUS and CI categories was the second-highest for this metapredictor (0.5105, *p* < 0.0001). The results of all comparisons, including statistical significance, are reported in Table 2.

To verify if the missense variants scores were influenced by proximity to splicing junctions, we examined the score distribution for 106 *NF1* missense variants with likely effects on splicing, since located ±3 nucleotides from splicing junctions. Of these 106 variants, the ClinVar classification was likely benign (1), VUS (85), CI (1) and leaning pathogenic (19). The score median of the 106 splicing variants was significantly higher than the median of LEANING BENIGN variants (VEST3 = 0.265, *p* < 0.0001; REVEL = 0.203, *p* = 0.0021; ClinPred = 0.5411, *p* < 0.0001) and significantly lower than the median of LEANING PATHOGENIC variants (VEST3 = −0.161, *p* < 0.0001; REVEL = −0.318, *p* = 0.0021; ClinPred = −0.0359, *p* < 0.0001). However, the removal of these variants from the dataset did not change either the score distribution, or the statistically significant differences between categories.

Overall, we observed a rather wide variability in the predictions scores for the three computational tools, with REVEL scores being generally the lowest and ClinPred showing the highest values. To further investigate the degree of relatedness between VEST3, REVEL and ClinPred scores, we performed a correlation analysis. In general, a loose correlation was present, with a decreasing correlation level of VEST3 vs. REVEL (*ρ* = 0.744, *p* < 0.0001), VEST3 vs. ClinPred (*ρ* = 0.699, *p* < 0.0001), and REVEL vs. ClinPred (*ρ* = 0.684, *p* < 0.0001) (Supplementary Figure S1).

**Figure 2:**
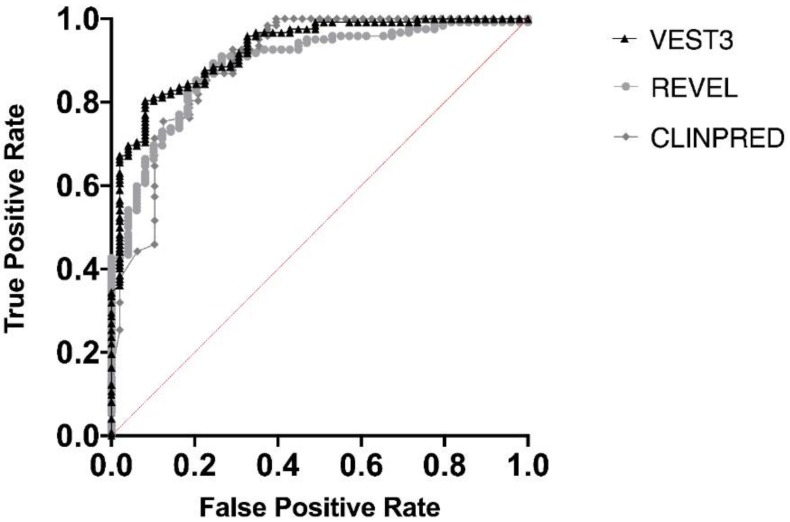
Overall performances of the three predictors on the *NF1* missense variants. The area under the curve (AUC) is reported for each predictor.

Next, we measured the area under the receiver operating characteristic (AUC) curves for all the three metapredictors. VEST had the highest AUC (0.9252) followed by ClinPred (AUC = 0.8939) and REVEL (AUC 0.8896) (Figure 2).

Supplementary Figure S2 shows the sensitivity and specificity values from the AUCs, corresponding to different predictors thresholds as cutoff for pathogenicity, which resulted quite different for VEST3, REVEL, and ClinPred. In Supplementary Table S2 the cutoff values best performing as the threshold for pathogenicity according to different performance indicators are reported. 

To investigate whether the prediction score values were correlated to functional domains within the neurofibromin protein, we examined the distribution of missense variant scores of different categories along the *NF1* coding sequence. Score values appeared non-randomly distributed with specific clustering more evident for REVEL scores (Supplementary Figure S3). Thus, we proceeded to verify if the score medians were significantly different among functional domains of the NF1 protein annotated at INTERPRO (https://www.ebi.ac.uk/interpro/beta/search/text/P21359/). 

**Figure 3:**
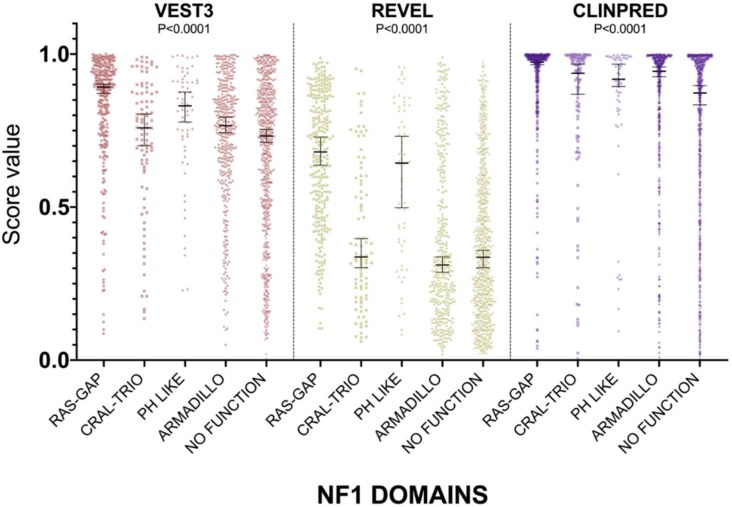
Prediction score values in the principal NF1 functional domains annotated at InterPRO (RAS-GTPase aa 1210–1549; CRAL-TRIO LIPID BINDING DOMAIN aa 1581–1726; PH-LIKE Pleckstrin homology domain aa 1727–1837; Armadillo type fold aa 1849–2676; NO FUNCTION all amino acid not comprised in the previous domains). Medians with 95% confidence intervals are reported. The P value of non-parametric ANOVA is reported for each predictor (Kruskal-Wallis test).

For all three computational tools, and for all domains, prediction scores were again not normally distributed (Supplementary Table S1). The non-parametric one way ANOVA demonstrated a statistically significant difference in score distribution among neurofibromin functional domains for all three metapredictors (VEST3 K-statistic = 107.3, REVEL K-statistic = 247.1, ClinPred K statistic = 80.5, and *p* < 0.0001 in all tests, Figure 3). 

In addition, the score medians in the RAS-GAP domain of neurofibromin were significantly higher compared to medians of scores in other functional domains and in protein regions where no functions have been assigned yet (VEST3 median = 0.892, REVEL median = 0.6805, ClinPred median = 0.974). The only exception was represented by the PH-Like pleckstrin domain where the score medians, for all metapredictors, were not significantly different, although slightly lower than medians in the RAS-GAP domain, (Table 3).

The PH-Like pleckstrin region showed the second-highest score median among INTERPRO annotated domains, again for both VEST (0.831) and REVEL (0.644), being significantly higher than score medians of all the other remaining regions. Similarly, even in ClinPred the PH-Like pleckstrin domain had significantly higher score median than the median in the non-functional region (0.918 vs. 0.873). In contrast, ClinPred demonstrated a rather diverse scores clustering, with score medians of variants in CRAL-TRIO and ARMADILLO domains significantly higher than score medians in the non-functional region (Table 3). 

### 2.2. Development of a Classifier for Classification Fine-Tuning

Since the score medians differences between the “LEANING BENIGN” and the “LEANING PATHOGENIC” categories of missense variants were highly significant for all metapredictors, we tested five different models to identify an accurate model for predicting variants pathogenicity potential based on scores distributions for the three computational methods. To this end, for each predictor and for each model, 80% of the subset of LEANING BENIGN and LEANIGN PATHOGENIC missense variants was used for training models, and 20% for validation. Accuracy on the validation set was used to measure each model performance. Next, we ran the best performing classifier (*LDA* for VEST3, accuracy = 0.85; *SVM* for REVEL, accuracy = 0.82; *kNN* for ClinPred, accuracy = 0.83) on a different subset of variants which included the validation set plus all VUS and CI variants. Medians, upper and lower 95% confidence intervals of scores from variants predicted as benign or pathogenic by these classifiers were used to proceed in variants’ reclassification. Specifically, missense variants were classified as LEANING BENIGN if they had scores below the upper 95% confidence interval of the predicted benign median, and as LEANING PATHOGENIC when the score was above the lower 95% confidence interval of the predicted pathogenic median. All variants with scores contained between the two boundaries were reclassified as VUS.

### 2.3. Reclassification Results after Training

**Figure 4:**
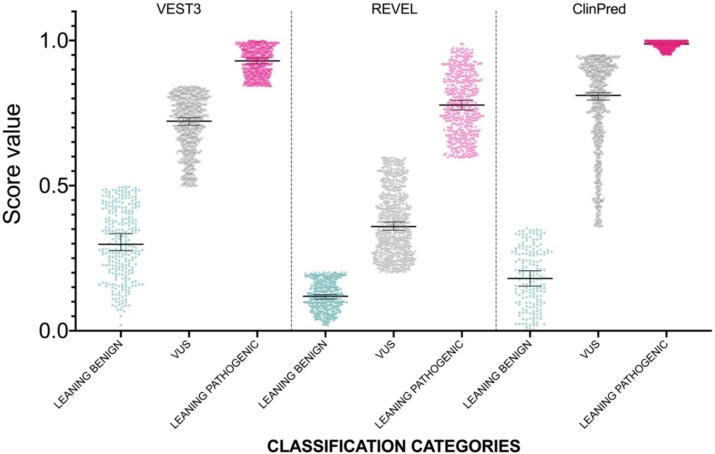
Prediction score values after reclassification of *NF1* missense variants in the three categories LEANING BENIGN, VUS, and LEANING PATHOGENIC. For each category, median and 95% confidence intervals are reported.

The distribution of newly classified variants’ scores showed a much better separation than before training for all the three predictors (Figure 4).

In detail, with VEST3 only 6 of 171 LEANING BENIGN and LEANING PATHOGENIC missense variants switched to the opposite category, while the number of VUS and CI variants was reduced from 1364 down to 707 (Table 4).

Similar results were obtained using REVEL scores for classification. In this case, 11 variants in total changed to the opposite category, while 777 variants were classified as VUS. Finally, for ClinPred, no variants previously classified as LEANING PATHOGENIC variants changed sign, whereas, 11 LEANING BENIGN were reclassified as LEANING PATHOGENIC. In addition, compared to VEST3 and REVEL, 107 and 202 more variants, respectively, were reclassified as LEANING PATHOGENIC in ClinPred (Table 4).

The previous *NF1* missense variants classification at ClinVar, and the novel classification for each predictor after training is presented in the Supplementary Table S3.

**Figure 5:**
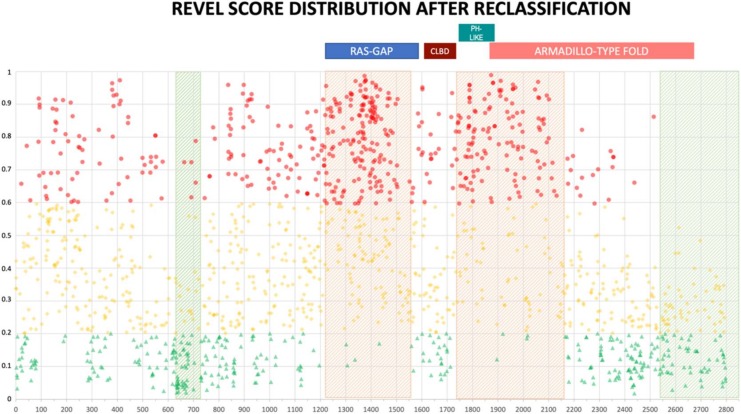
REVEL prediction score values distribution throughout the NF1 protein. Above, the location of the four NF1 InterPRO domains (RAS-GTPase aa 1210–1549; CRAL-TRIO LIPID BINDING DOMAIN aa 1581–1726; PH-LIKE Pleckstrin homology domain aa 1727–1837; Armadillo type fold aa 1849–2676). Green triangles are LEANING BENIGN scores, yellow diamonds VUS scores, red circles LEANING PATHOGENIC scores. Shaded in red are regions with clustering of LEANING PATHOGENIC variants with high prediction scores (aa 1200–1440, and 1725–1250), while green shading indicates regions where no or few LEANING PATHOGENIC variants are present (aa 2530–2849 and 600–700, respectively).

The distribution of reclassified variants along the neurofibromin protein reinforced the non-random distribution we observed before. In fact, no newly classified LEANING PATHOGENIC variants were present beyond amino acid 2550 for REVEL (2700 for VEST3) (Figure 5 and Supplementary Figure S4). Once more, REVEL demonstrated the most skewed distribution with only two LEANING PATHOGENIC variants after amino acid 2400 and three between amino acids 600-700. Also, in these regions clustered several LEANING BENIGN variants (94 and 22, respectively, 33% of all LEANING BENIGN variants overall, Figure 5).

In contrast, ClinPred did not show a similar uneven distribution of variants along neurofibromin (Supplementary Figure S5). Further, non-functional regions of neurofibromin which showed clustering of missense variants with low pathogenicity scores presented also looser evolutionary conservation (Supplementary Figure S6).

## 3. Discussion

With the advent of massive sequencing technologies, the number of variants identified, while screening disease-causing genes has steeply increased. Thus, variant interpretation represents the most challenging and time-consuming step in the NGS workflow analyses [46]. Correct identification of disease-causing variants is fundamental in precision medicine, and several prediction tools are currently available to aid in variant interpretation. However, some mendelian diseases may exhibit more complex genetics being caused by mutations having both subtle and/or additive effects responsible for pathogenicity. Here, we aimed to fine-tune prediction tools at a single gene level and improve predictions accuracy, while simplifying the variant interpretation process. In fact, several recent studies have compared individual predictors performance on large heterogeneous databases of variants [32,33,47]. However, their performances at single gene level have not been thoroughly investigated. 

We used the *NF1* variant spectrum, since neurofibromatosis type 1 clinical diagnosis is usually suspected in early years of life when canonical symptoms may not be present or fully developed [48]. The later onset of NF1 clinical features, in combination with the 50% rate of *de novo* non-segregating mutations, makes variant interpretation particularly challenging. Indeed, nearly half of variants deposited at ClinVar, and almost 90% of the missense changes are of uncertain significance. This scenario is further complicated by the milder phenotype observed in patients with either missense variants and/or in frame deletions and insertions [49,50,51]. 

Our analysis focused on investigating and comparing the performances of three recently developed prediction tools that have been tested on large datasets and less frequently on single disease-causing genes. First, we noticed that *NF1* missense variants scores for the three predictors VEST3, REVEL and ClinPred were loosely correlated. ClinPred scores were higher on average than corresponding VEST3 and REVEL scores. Despite the poor correlation, the three computational methods had comparable performances applied to the *NF1* missense variant dataset. It is possible that different criteria used to categorize variants, especially those classified as benign, based on different allelic frequencies for filtering variants, or using untested score values as cutoffs between benign and pathogenic variants may account for these differences.

Indeed, the score values performing better as the cutoff for pathogenicity were rather diverse between the three predictors, and unlike values originally reported [30,31]. These results suggest the need to tailor gene-specific thresholds to achieve a more accurate pathogenicity assessment. The utility of establishing gene-specific thresholds has been already reported previously. Also, several computational methods, including REVEL and VEST3, have been demonstrated reliable even when tested on unbalanced datasets (i.e., unequal number of benign and pathogenic variants) [32,52]. It should be said that selecting a precise cutoff for pathogenicity is a subjective choice. In fact, individual users may want to customize their own acceptable tradeoff between putative pathogenic variants identified, and the rate of false positive predictions.

In our analyses, we found no significant differences between scores of variants with a “likely” assertion and those with a definitive “benign” or “pathogenic” classification. For this reason, we merged the “likely” and “definite” classes into a single category that we named “LEANING”. The same approach has been demonstrated, not affecting performances of several prediction tools [32]. Moreover, from a clinical point of view, no different actions are usually taken in patients’ management when gene testing results reveal a “likely” variant rather than a “definite”. 

Next, we aimed to reduce the number of VUS variants (88% of the dataset) with the same strategy already utilized to improve *MEFV* gene variants classification in Familial Mediterranean Fever (FMF), a disease where clinical misdiagnosis can be as frequent as in NF1 [53].

We obtained a much sharper separation between the LEANING BENIGN, VUS, and LEANING PATHOGENIC categories scores for all the three prediction tools. Further, we were able to halve the number of VUS variants (down to 51.5% with ClinPred, 52% with REVEL, and 52.6% with VEST3), while only a marginal number of variants switched to the opposite classification category (6 in VEST3, 11 in REVEL and in ClinPred).

We noticed that while in the original reports recommended thresholds were not provided (VEST3 and REVEL) or a 0.5 value had been used (ClinPred), gene-specific cutoffs can be variable and optimized for a better predictions’ accuracy. This approach may be extremely useful in a disease context, such as NF1, where clinical information could be missing or weakly supportive. In previous studies, a combination of different prediction tools has been suggested [47]. However, we observed a weak correlation between the three metapredictors scores of *NF1* missense variants. Hence, we hypothesize that combining different computational methods may lead to a loss of information which may be critical at least for assessing pathogenicity of specific *NF1* variants.

A recent study investigated the performances of several predictors, including VEST3 and REVEL, on different *NF1* datasets with pathogenic variants extracted from the Leiden Open Variation Database (LOVD) [54]. The authors of this paper considered benign any variant with an allelic frequency above 0 in the 1000Genomes Project, The Exome Aggregation Consortium (ExAC), the Genome Aggregation Database (gnomAD), and not previously reported as pathogenic [54]. Further, *NF1* variants affecting splicing junctions were also used, and the localization to the NF1 armadillo domain was demonstrated to increase the likelihood for a variant of being pathogenic. In our work, on a different dataset (all *NF1* missense variants deposited at ClinVar), we evidenced a higher median score difference between the LEANING BENIGN and LEANING PATHOGENIC variants, for both VEST3 and REVEL (0.42 vs. 0.19, and 0.523 vs. 0.24, respectively) compared to the one reported by Isakov et al. [54]. In addition, our variant reclassification evidenced a preferential clustering, in the neurofibromin RAS-GAP and PH-LIKE domains, of REVEL leaning pathogenic scores. In contrast, no variants with pathogenic scores were present in the last 300 amino acid of the protein and in a narrow region of 50 residues (600–650). A similar, albeit less marked non-random distribution was observed for VEST3 scores, while ClinPred scores were rather evenly spread along neurofibromin. Overall, variant reclassification resulted in a sharp separation of prediction scores throughout the whole coding sequence of NF1 protein for all three metapredictors. Interestingly, non-functional NF1 regions with few or no variants predicted as pathogenic, had also a looser evolutionary conservation compared to the rest of unannotated portions of NF1.

The validity of the approach presented here, is demonstrated by scores of missense variants located in NF1 amino acid 844-848. These variants have been associated prevalently to superficial plexiform neurofibromas, symptomatic spinal neurofibromas, and a higher risk of developing malignancies [51].

Twelve variants in this interval were listed at ClinVar, and classified as pathogenic (7), with conflicting interpretation (1) or VUS (4). After reclassification, 10/12 variants were in the LEANING PATHOGENIC category for both VEST3 and ClinPred (8 for REVEL), and only 2 were reclassified as VUS (4 in REVEL). More in detail, the two variants Leu847Val and Leu847Phe classified as VUS by both VEST3 and ClinPred were not among those responsible for the severe NF1 manifestations reported by Koczkowska et al. [51]. In contrast, variants Leu847Pro and Leu847Arg, present in 78 individuals with this severe phenotype were scored as LEANING PATHOGENIC by all three predictors. Similarly, the Arg1809Cys, Arg1809Leu, Arg1809Pro, Arg1809Ser, and Arg1809Gly, responsible for a peculiar NF1 phenotype with the frequent association to short stature and pulmonic stenosis, but lacking cutaneous and plexiform neurofibromas [55], had all very high scores computed by the three algorithms (average 0.97). Lastly, one of the only three variants predicted as pathogenic by REVEL in the NF1 region between amino acids 600–700 (Leu691Arg), was found in a six years-old child with >6 café-au-lait macules and brain neurofibromatosis [56]. This variant also had high pathogenic scores computed by both VEST3 and REVEL. Hence, it appears that VEST3, REVEL and ClinPred quite reliably predict the pathogenic potential of variants even when associated with the non-classical form of NF1.

A possible issue of analyses, such as those performed in this work, may derive from circularity in data used [57]. However, both VEST3 and REVEL were originally trained on a Human Gene Mutation Database (HGMD) dataset different from the one we used. The ClinPred original training dataset included 11082 benign and pathogenic missense variants downloaded from ClinVar on January 2016, with review status of “criteria provided” from submitter or “reviewed by expert panel,” and not added to ClinVar before January 2013. Only 12 variants fulfilling these criteria were present in our training dataset and were already submitted to ClinVar prior to January 2016. Thus, our initial dataset of *NF1* benign and pathogenic variants (*n* = 171) might have, at best, overlapped with ClinPred training set for 0.1% (12/11082). This makes type 1 circularity problems for ClinPred results highly unlikely. Since in our *NF1* variant sample, benign and pathogenic variants were rather even in number, a type 2 circularity should be less likely. Thus, while we cannot exclude that some circularity problem may remain, the balanced gene-specific subset we analyzed should limit its influence on our results.

The 2015 ACMG/AMP guidelines for variant classification remains the mainstay and an invaluable resource in variant interpretation. In this work, we present evidence that the prediction scores of *NF1* variants classified as “benign” or “likely benign”, and “likely pathogenic”, or “pathogenic” do not differ significantly. In NF1, some of the ACMG/AMP classification criteria cannot be used, and patients’ management does not usually differ when variants fall into” likely” rather than “definite” categories.

Thus, specifically for NF1, we propose a simpler classification that may help in a more streamlined adoption of proper clinical management of NF1 patients after genetic testing.

## 4. Materials and Methods

### 4.1. Dataset of Missense Variants

The analyzed dataset was represented by all the missense variants for the *NF1* gene (NM_000267.3, NM_001042492.2, both transcripts are indifferently used in ClinVar) listed at ClinVar [58]. Because only eight missense variants classified as either “benign” and “likely benign” were present in the *NF1* ClinVar database, we supplemented these categories with 8 *NF1* missense variants classified as benign or likely benign and uniquely present at the LOVD-*NF1* database [59]. In addition, 33 further *NF1* missense variants listed as VUS in ClinVar were reclassified as benign or likely benign in agreement with their classification at LOVD. All databases were last accessed on 31 May 2019. In total, 1585 *NF1* missense variants were analyzed. Missense variants whose classification in ClinVar was not provided were excluded from the analysis.

### 4.2. Statistical Analyses

We extracted the VEST3 prediction scores uploading all the variants in the analyzed dataset at the server website [60]. For both REVEL and ClinPred prediction scores are precomputed for each sequence position on the hg19 (GRCh37) human genome build, and available at REVEL [61] and ClinPred [62], respectively. The prediction scores for all *NF1* missense variants analyzed, their initial classification at ClinVar, and proposed novel classification are available in the Supplementary Table S3. Means, medians, SD, and the 95% confidence interval of the medians were assessed for each ClinVar category.

Normality of the scores’ distribution for all ClinVar classes was initially assessed with the Shapiro-Wilk test. Next, the differences between “likely benign” vs. “benign”, and “likely pathogenic” vs. “likely pathogenic/pathogenic” vs. “pathogenic” were assessed with Mann-Whitney test and Kruskal-Wallis test of non-parametric ANOVA, respectively.

After the merging into “LEANING BENIGN” and “LEANING PATHOGENIC” novel categories, the normality of score distribution for all categories and predictors was again checked with Shapiro-Wilk test. The statistical significance of score distribution differences between categories was evaluated by non-parametric One-Way ANOVA with Kruskal-Wallis test. Next, pairwise correlations between categories were assessed with Dunn’s test corrected for False Discovery Rate by Benjamini-Hochberg method. To evaluate the association between VEST3, REVEL and ClinPred scores, the non-parametric Spearman’s correlation coefficient was used. All tests of statistical significance were two-tailed, and *P*-values were considered statistically significant if <0.05.

The measures used for predictors’ performance evaluation and to build the ROC curve are reported in Supplementary File S4.

All statistical analyses were performed using Prism v.8.3.0 (GraphPad Software, LLC, San Diego, CA, USA)

### 4.3. Five Model Classification

Five different models were tested to perform classification, specifically: Linear Discriminant Analysis (*LDA*), Classification and Regression Trees (*CART*), k-Nearest Neighbors (*kNN*), Support Vector Machines (*SVM*) with a linear kernel, and Random Forest (*RF*). This set of algorithms was chosen as it represents a good combination of linear and non-linear models to test. The dataset used for training models, was represented for each predictor by the “LEANING BENIGN” (*n* = 49) and “LEANING PATHOGENIC” (*n* = 122) variants present in our dataset. Therefore, the resulting 171 variants dataset was split into two parts, 80% was used to train the models through 10-fold cross validation, and 20% was used as a validation test set. All models were weighted using the “accuracy” metric. The parameters used for training models in Caret were: Method = “repeatedcv”; number = 10; repeats = 5; classProbs = T; set.seed (7). Computations were performed in R (version 3.6.1) using the Caret (v.6.0-84) package.

## 5. Conclusions

The focus of our study was to improve *NF1* missense variants interpretation and classification, considering that 66.2% (1045/1579) of missense variants listed at ClinVar were reported by single submitters with little supportive evidence for classification criteria. Thus, the results presented in this article may serve as a useful resource for laboratories involved in NF1 genetic testing, as well as an aid in developing variant interpretation guidelines for additional gene-disease systems. 

## Figures and Tables

**Table 1:** Descriptive statistics of ClinVar *NF1* variants prediction scores by VEST3, REVEL, CLINPRED.

	*VEST3*				*REVEL*				*ClinPred*			
	**LB**	**VUS**	**CI**	LP	**LB**	**VUS**	**CI**	LP	**LB**	**VUS**	**CI**	**LP**
**Number of Values**	49	1363	50	122	49	1360	50	122	48	1364	50	122
**Minimum**	0.02	0.067	0.09	0.347	0.038	0.018	0.021	0.026	0.005	0.019	0.004	0.596
**25% Percentile**	0.2435	0.584	0.434	0.8728	0.1145	0.2103	0.1585	0.5203	0.1318	0.6915	0.124	0.9828
**Median**	0.528	0.77	0.707	0.948	0.216	0.381	0.274	0.7395	0.394	0.914	0.4035	0.993
**75% Percentile**	0.742	0.885	0.9558	0.989	0.365	0.645	0.683	0.8848	0.944	0.9810	0.9823	0.998
**Maximum**	0.978	1.000	0.998	0.9990	0.785	0.987	0.909	0.9890	0.998	1.000	1.000	1.000
**95% CI of Median**												
**Actual Confidence Level**	95.56%	95.00%	96.72%	96.31%	95.56%	95.00%	96.72%	96.31%	97.07%	95.00%	96.72%	96.31%
**Lower Confidence Limit**	0.423	0.755	0.558	0.936	0.168	0.356	0.216	0.681	0.176	0.905	0.156	0.991
**Upper Confidence Limit**	0.67	0.783	0.815	0.972	0.298	0.401	0.494	0.809	0.828	0.927	0.889	0.995
**Coefficient of Variation**	51.5%	32.39%	43.51%	12.93%	73.59%	60.89%	72.6%	34.83%	78.04%	31.97%	80.05%	6.651%

LB = Leaning Benign; VUS = Variant of uncertain significance; CI = Conflicting Interpretation; LP = Leaning Pathogenic.

**Table 2:** Pairwise comparison between scores of variants with different classification at ClinVar. Results of Dunn’s test corrected for multiple comparisons with the Benjamini-Hochberg method are shown. In bold and italics are highlighted results of pairwise comparisons between the LEANING BENIGN and LEANING PATHOGENIC categories showing the widest medians difference for all the three predictors.

		Medians Difference	Adjusted *p* Value
**VEST3**	LEANING BENIGN vs. VUS	−0.242	<0.0001
	LEANING BENIGN vs. CI	−0.179	0.0022
	***LEANING BENIGN* vs. *LEANING PATHOGENIC***	***−0.42***	***<0.0001***
	VUS vs. CI	0.063	0.578
	VUS vs. LEANING PATHOGENIC	−0.178	<0.0001
	CI vs. LEANING PATHOGENIC	−0.241	<0.0001
**REVEL**	LEANING BENIGN vs. VUS	−0.165	<0.0001
	LEANING BENIGN vs. CI	−0.058	0.0284
	***LEANING BENIGN* vs. *LEANING PATHOGENIC***	***−0.5235***	***<0.0001***
	VUS vs. CI	0.107	0.3313
	VUS vs. LEANING PATHOGENIC	−0.3585	<0.0001
	CI vs. LEANING PATHOGENIC	−0.4655	<0.0001
**ClinPred**	LEANING BENIGN vs. VUS	−0.52	<0.0001
	LEANING BENIGN vs. CI	−0.0095	0.3826
	***LEANING BENIGN* vs. *LEANING PATHOGENIC***	***−0.599***	***0.0004***
	VUS vs. CI	0.5105	<0.0001
	VUS vs. LEANING PATHOGENIC	−0.079	<0.0001
	CI vs. LEANING PATHOGENIC	−0.59	<0.0001

**Table 3:** Pairwise comparison of score distribution in the NF1 protein domain. Results of Dunn’s test corrected for multiple comparisons with the Benjamini-Hochberg method are shown.

VEST3 Scores Score Comparison INTER DOMAINS	Median Difference	*Adjusted p* Value
RAS-GAP vs. CRAL-TRIO	0.133	<0.0001
RAS-GAP vs. PH LIKE	0.061	0.1102
RAS-GAP vs. ARMADILLO	0.126	<0.0001
RAS-GAP vs. NO FUNCTION	0.1595	<0.0001
CRAL-TRIO vs. PH LIKE	−0.072	0.0498
CRAL-TRIO vs. ARMADILLO	−0.007	0.7220
CRAL-TRIO vs. NO FUNCTION	0.0265	0.3105
PH LIKE vs. ARMADILLO	0.065	0.0405
PH LIKE vs. NO FUNCTION	0.0985	0.0009
ARMADILLO vs. NO FUNCTION	0.0335	0.0174
**REVEL Scores Score Comparison INTER DOMAINS**	**Median Difference**	***p* Value**
RAS-GAP vs. CRAL-TRIO	0.3435	<0.0001
RAS-GAP vs. PH LIKE	0.0365	0.1087
RAS-GAP vs. ARMADILLO	0.3695	<0.0001
RAS-GAP vs. NO FUNCTION	0.3445	<0.0001
CRAL-TRIO vs. PH LIKE	−0.307	0.0001
CRAL-TRIO vs. ARMADILLO	0.026	0.5751
CRAL-TRIO vs. NO FUNCTION	0.001	0.3637
PH LIKE vs. ARMADILLO	0.333	<0.0001
PH LIKE vs. NO FUNCTION	0.308	<0.0001
ARMADILLO vs. NO FUNCTION	−0.025	0.4453
**ClinPred Scores Score Comparison INTER DOMAINS**	**Median Difference**	***p* Value**
RAS-GAP vs. CRAL-TRIO	0.0365	0.013
RAS-GAP vs. PH LIKE	0.056	0.066
RAS-GAP vs. ARMADILLO	0.03	0.0002
RAS-GAP vs. NO FUNCTION	0.101	<0.0001
CRAL-TRIO vs. PH LIKE	0.0195	0.8558
CRAL-TRIO vs. ARMADILLO	−0.0065	0.8558
CRAL-TRIO vs. NO FUNCTION	0.0645	0.0283
PH LIKE vs. ARMADILLO	−0.026	0.8558
PH LIKE vs. NO FUNCTION	0.045	0.0253
ARMADILLO vs. NO FUNCTION	0.071	<0.0001

**Table 4:** Classification categories of variants before and after training predictors. # = number.

METAPREDICTOR	Variant Classification at ClinVar	# of Variants Reclassified as *Leaning Benign*	# of Variants Reclassified as *VOUS*	# of Variants Reclassified as *Leaning Pathogenic*
**VEST3**	B, LB, B/LB	20	24	5
	VUS	251	641	471
	CI	14	19	17
	LP, LP/P, P	1	23	98
	***TOTAL***	***286***	***707***	***591***
**REVEL**	B, LB, B/LB	23	21	5
	VUS	322	644	394
	CI	17	19	14
	LP, LP/P, P	6	33	83
	***TOTAL***	***368***	***717***	***496***
**CLINPRED**	B, LB, B/LB	23	14	11
	VUS	136	659	569
	CI	24	12	14
	LP, LP/P, P	0	18	104
	***TOTAL***	***183***	***703***	***698***

## Supplementary Materials

Supplementary materials can be found at https://www.mdpi.com/1422-0067/21/3/721/s1.

## Abbreviations

NGS	Next Generation Sequencing
DCG	Disease-causing gene
ACMG	American College of Medical Genetics
AMP	Association for Molecular Pathology
VUS	Variant of uncertain significance
NF1	Neurofibromatosis type 1
GAP	GTPase activating protein
CI	Variant with Conflicting Interpretation
LOVD	Leiden Open Variation Database
HGMD	Human Gene Mutation Database
