# Supplementary material for: In-silico Analysis of NF1 Missense Variants in ClinVar: Translating Variant Predictions into Variant Interpretation and Classification
Source: Int J Mol Sci. 2020 Jan 22;21(3):721. doi: 10.3390/ijms21030721 (PMC7037781; doi:10.3390/ijms21030721)
Supplement: Supplementary file 1 [file ijms-21-00721-s001.zip › ijms-614345-supplementary files/ijms-614345-Supplementary figures.docx]

**
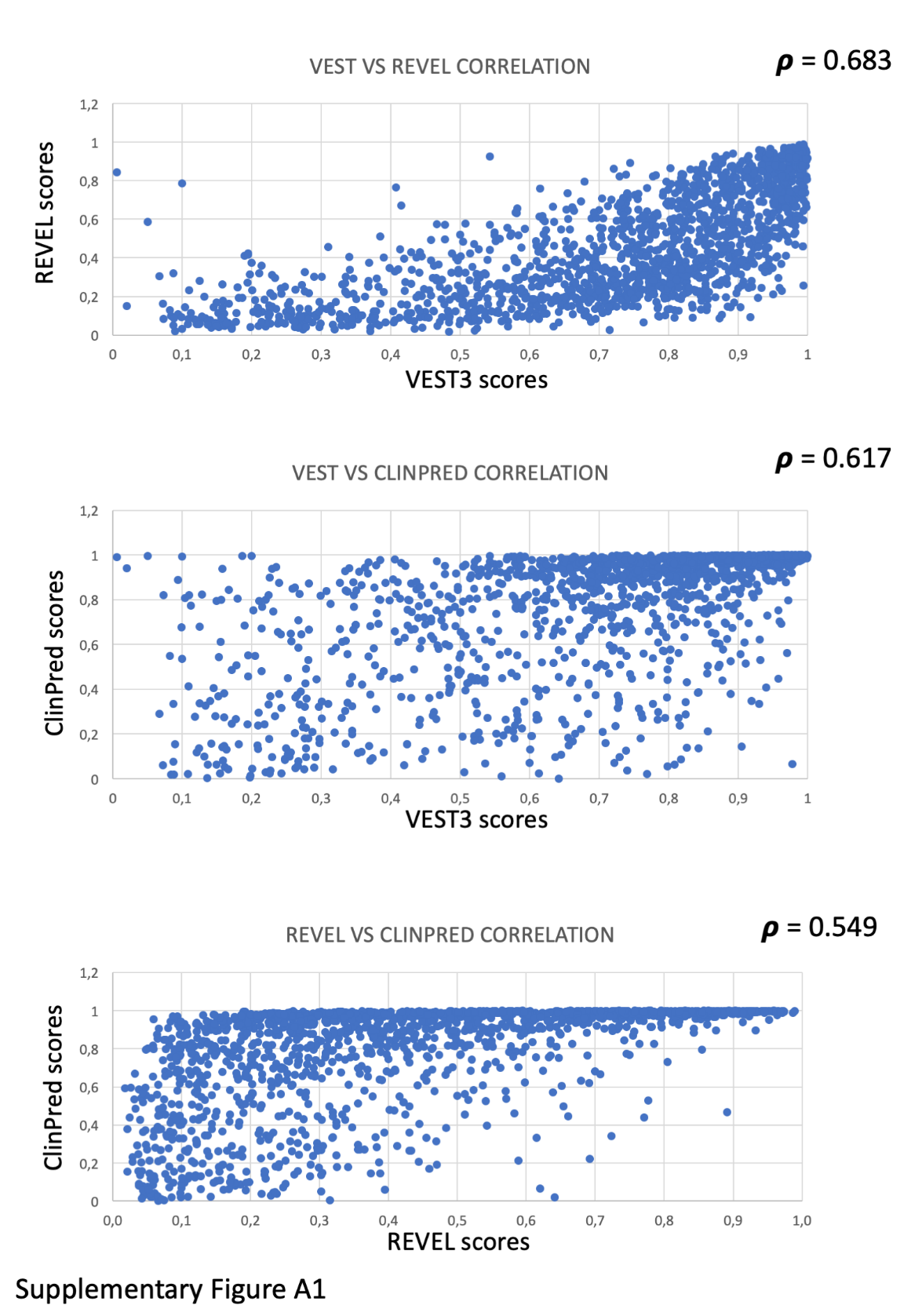
**

**Supplementary Figure S1. Correlation analysis of VEST3, REVEL and ClinPred scores.** The Pearson non-parametric correlation values (*ρ*) are indicated.


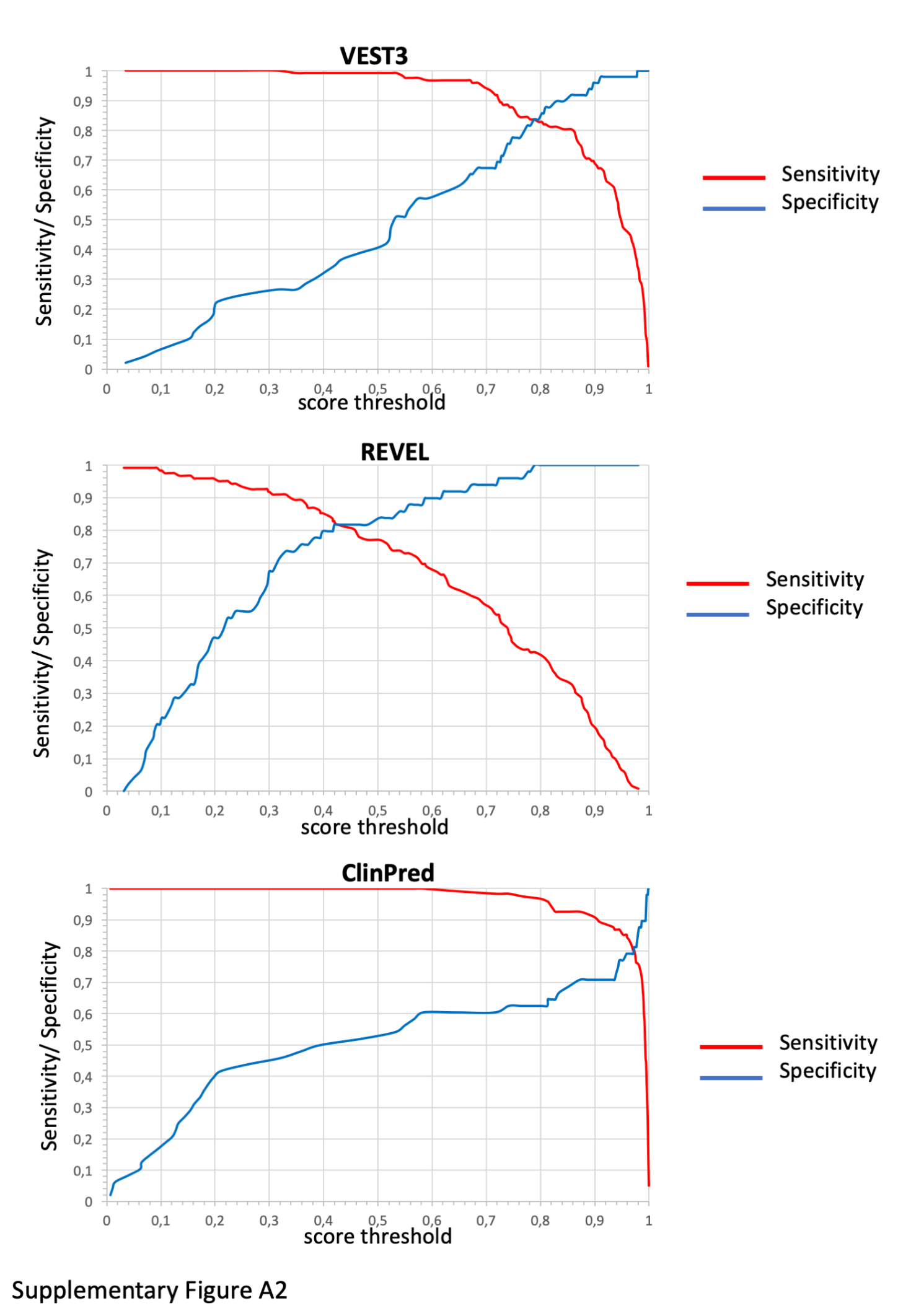


**Supplementary Figure S2. Sensitivity and specificity for different score thresholds for the three metapredictor.** The 49 LEANING BENIGN and 122 LEANING PATHOGENIC prediction scores were used. For each cutoff threshold value, true positives (TP) were considered LEANING PATHOGENIC variants with prediction score above cutoffs, true negatives (TN) LEANING BENIGN variants with prediction score below cutoffs, false positives (FP) LEANING BENIGN variants with prediction scores above cutoffs, and false negatives (FN) LEANING PATHOGENIC variants with prediction scores below cutoffs.


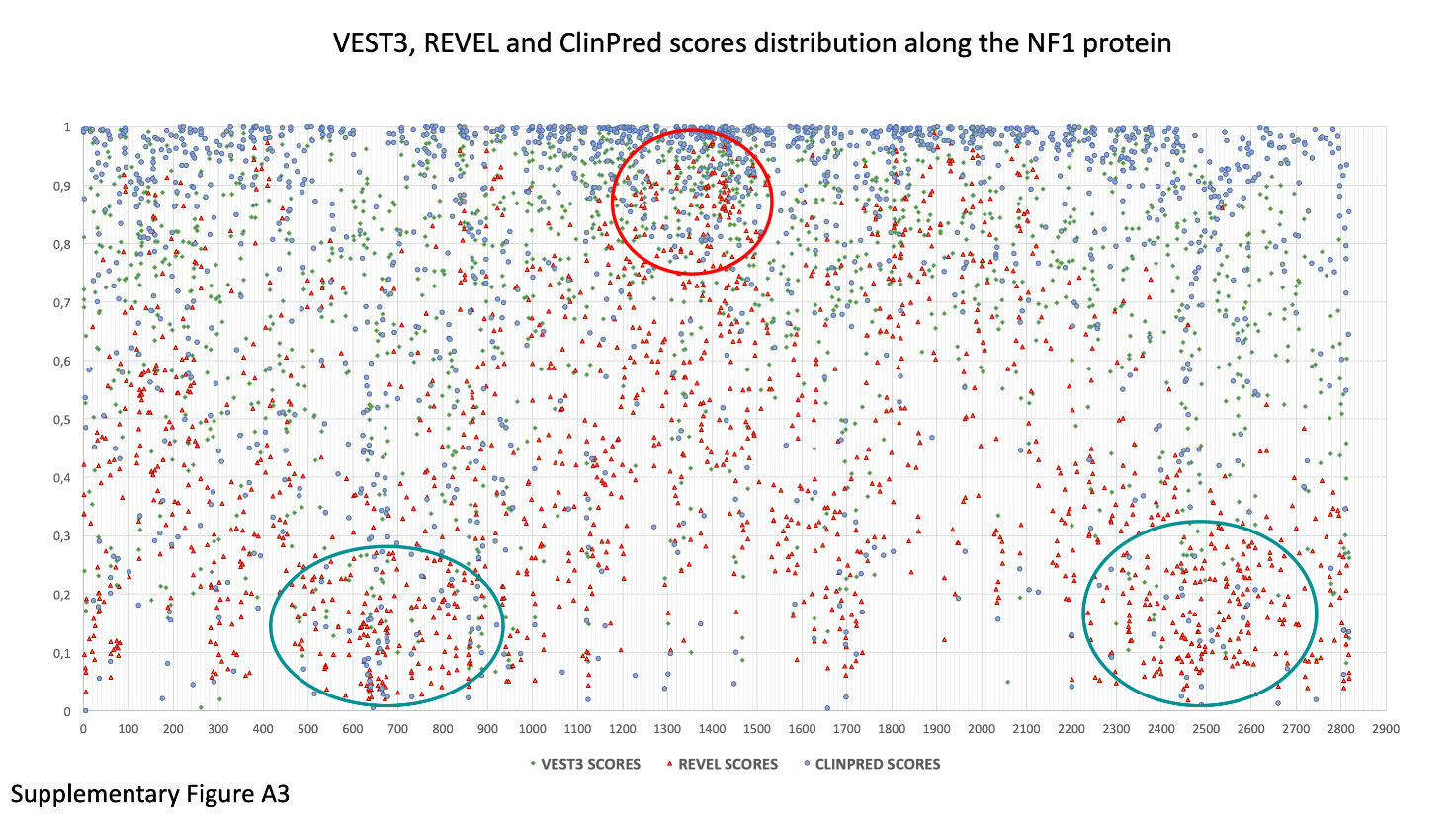


**Supplementary Figure S3. Distribution of VEST3, REVEL, and ClinPred prediction scores throughout the NF1 protein.** Circled in red is a region with clustering of REVEL pathogenic scores, while in green are highlighted regions with clustering of REVEL low prediction scores.


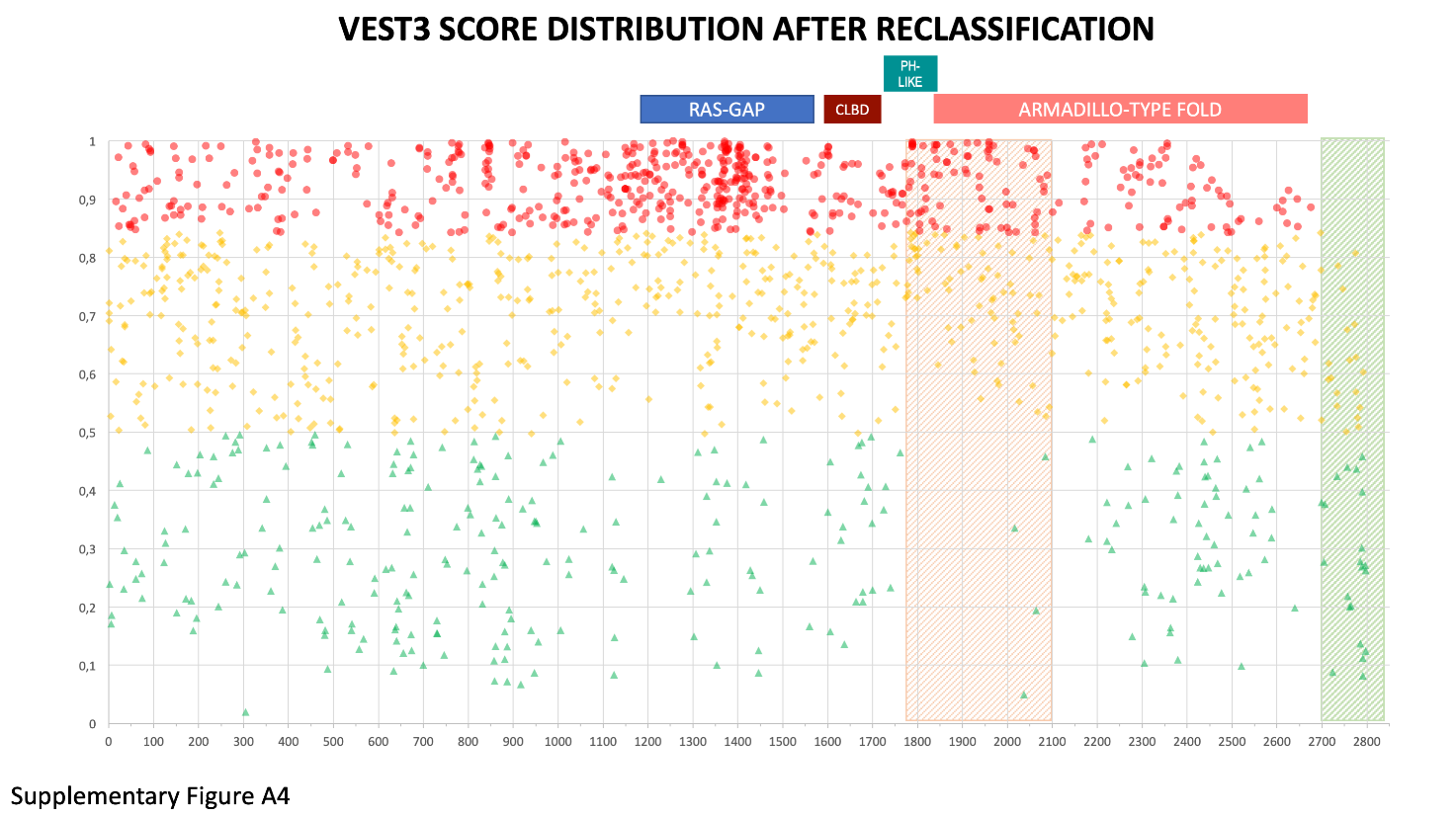


**Supplementary Figure S4. Distribution of VEST3 prediction scores throughout the NF1 protein after reclassification of variants.** The red shading indicates the NF1 region with accumulation of variants reclassified as LEANING PATHOGENIC (red dots) and with few variants reclassified as LEANING BENIGN (green triangles). Yellow diamonds show variants reclassified as VUS. The green shading highlights the C-terminal region of NF1 protein where no variants are predicted to be pathogenic. On Y-axis prediction score value.


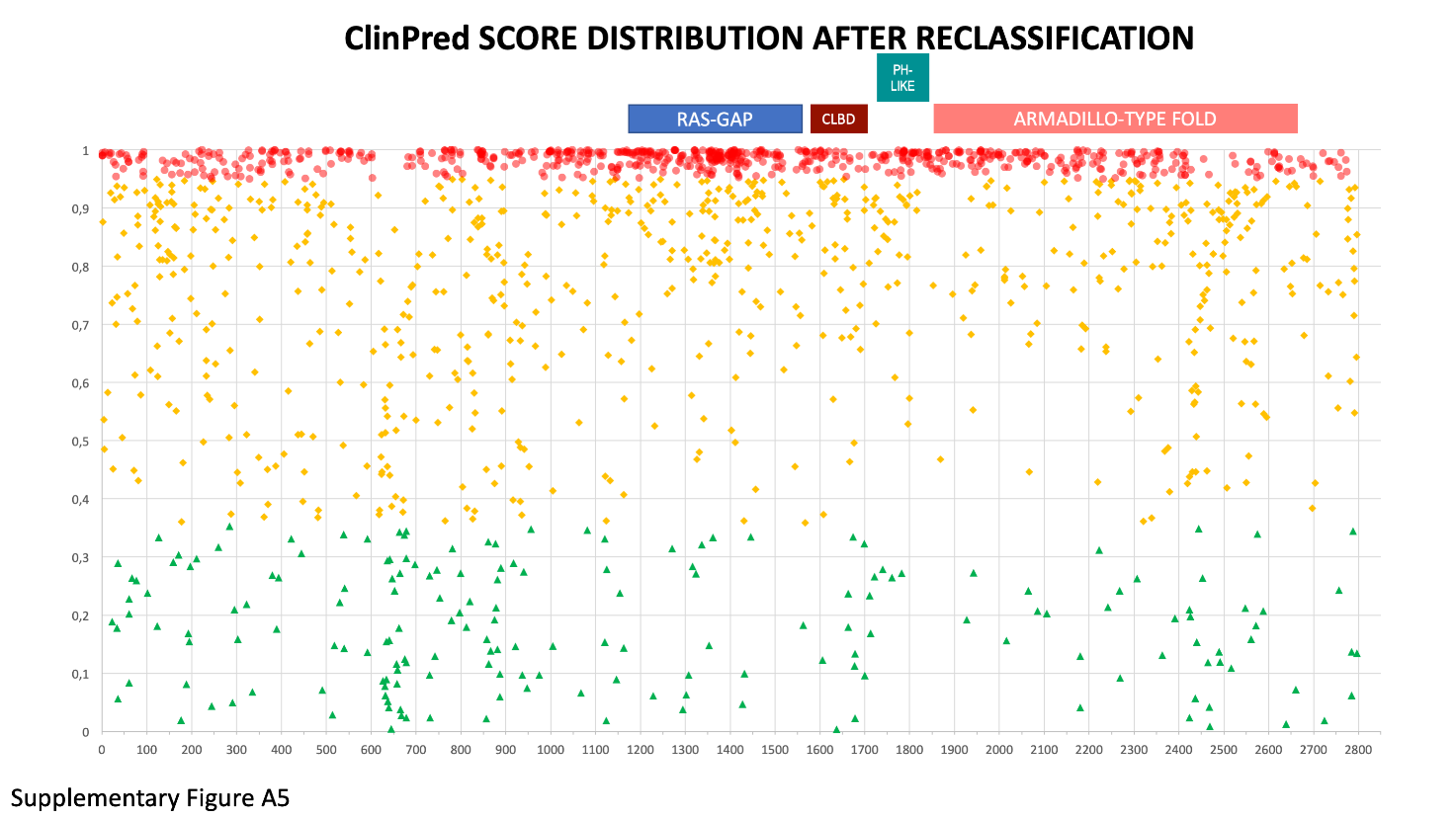


**Supplementary Figure S5.** **Distribution of ClinPred prediction scores throughout the NF1 protein after reclassification of variants.**

Distribution of variants classified as LEANING PATHOGENIC (red dots), as VUS (yellow diamonds) and as LEANING BENIGN (green triangles). On Y-axis prediction score value.


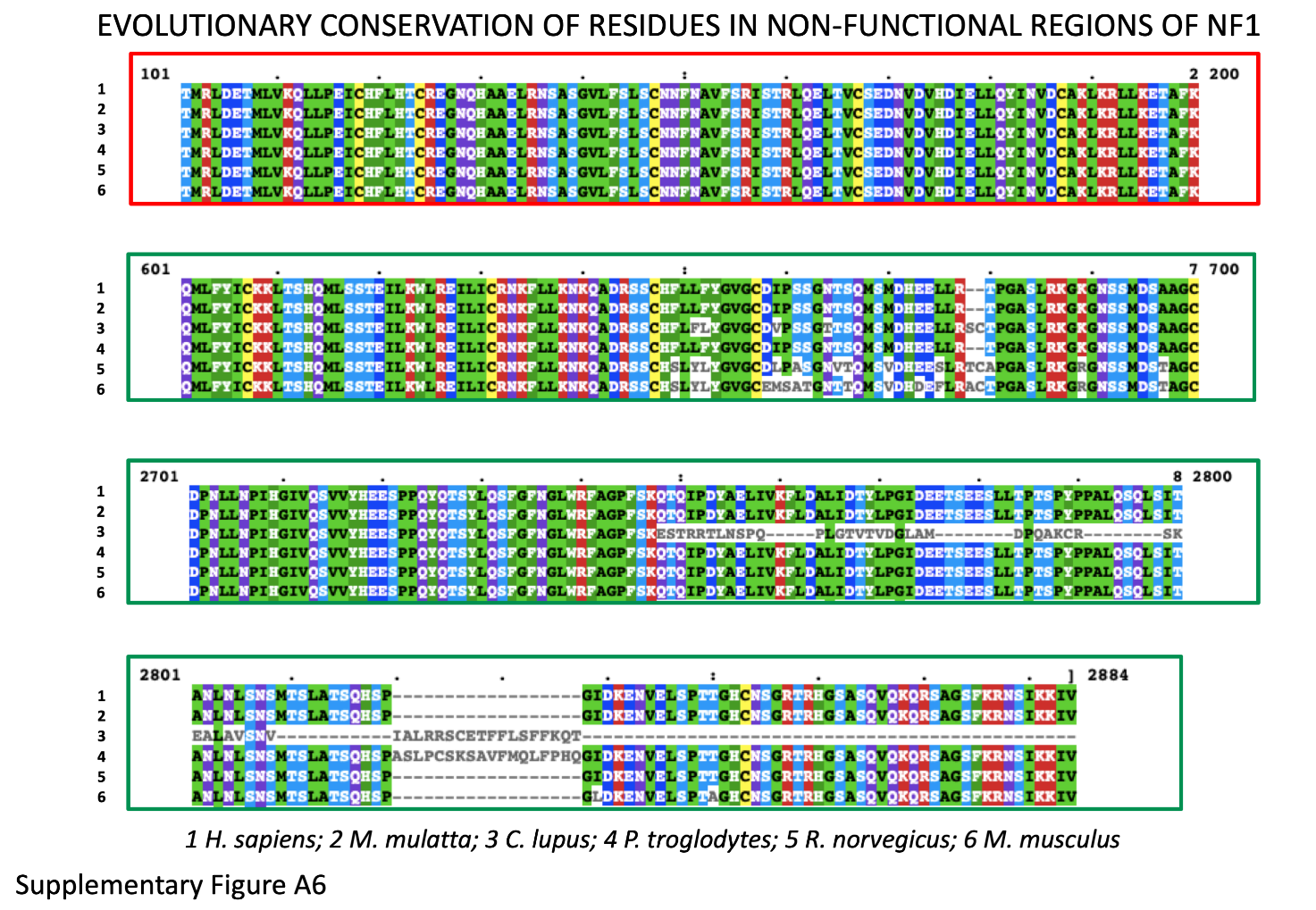


**Supplementary Figure S6. Evolutionary conservation of amino acid residues.** The red box indicates an NF1 region (amino acids 100-200) with underrepresentation of LEANING BENIGN variants. The green boxes show NF1 regions with no or limited number of LEANING PATHOGENIC variants (amino acid residues 600-700, second panel from top; amino acid residues 2700-2884 bottom two panels).
