# Supplementary material for: In-silico Analysis of NF1 Missense Variants in ClinVar: Translating Variant Predictions into Variant Interpretation and Classification
Source: Int J Mol Sci. 2020 Jan 22;21(3):721. doi: 10.3390/ijms21030721 (PMC7037781; doi:10.3390/ijms21030721)
Supplement: Supplementary file 1 [file ijms-21-00721-s001.zip › ijms-614345-supplementary files/SUPPLEMENTARY FILE S4.docx]

**SUPPLEMENTARY FILE S4**

**Measures used for performance evaluations.**

To assess the performance of the three different predictors’ score values, the following parameters were used where TP= true positive (LEANING PATHOGENIC variants with score above cutoff); TN= true negative (LEANING BENIGN variants with score below cutoffs); FP= false positive (LEANING BENIGN variants with score above cutoffs); FN= false negative (LEANING PATHOGENIC variants with score below cutoff).

**Sensitivity**

TP$\frac{}{}$TP + FN

**Specificity**

TN$\frac{}{}$TN + FP

**Accuracy**

TP + TN$\frac{}{}$ TP + FP + TN + FN

**Matthews correlation coefficient (MCC)**

TP × TN − FP × FN$\frac{}{}\sqrt{(TP + FP) \times(TP + FN) \times(TN + FP) \times(TN + FN)}$

**Youden's J index**

Sensitivity + Specificity – 1
