# Supplementary material for: In-silico Analysis of NF1 Missense Variants in ClinVar: Translating Variant Predictions into Variant Interpretation and Classification
Source: Int J Mol Sci. 2020 Jan 22;21(3):721. doi: 10.3390/ijms21030721 (PMC7037781; doi:10.3390/ijms21030721)
Supplement: Supplementary file 1 [file ijms-21-00721-s001.zip › ijms-614345-supplementary files/Supplementary Table S1.docx]

**Supplementary Table S1**

Results of the Shapiro-Wilk test for normal distribution in all categories studied

| **METAPREDICTOR** | **CATEGORIES** | **TEST STATISTIC (W)** | **P VALUE** |
| --- | --- | --- | --- |
| VEST3 | Leaning Benign | 0.95 | 0.03 |
|  | VOUS | 0.90 | <0.0001 |
|  | CI | 0.91 | 0.0012 |
|  | Leaning Pathogenic | 0.76 | <0.0001 |
| REVEL | Leaning Benign | 0.88 | 0.0002 |
|  | VOUS | 0.95 | <0.0001 |
|  | CI | 0.88 | 0.0001 |
|  | Leaning Pathogenic | 0.92 | <0.0001 |
| ClinPred | Leaning Benign | 0.83 | <0.0001 |
|  | VOUS | 0.77 | <0.0001 |
|  | CI | 0.81 | <0.0001 |
|  | Leaning Pathogenic | 0.5 | <0.0001 |
| DOMAIN ANALYSIS | | | |
| VEST3 | RAS-GAP | 0.79 | <0.0001 |
|  | CRAL-TRIO | 0.9 | <0.0001 |
|  | PH LIKE | 0.87 | <0.0001 |
|  | ARMADILLO | 0.92 | <0.0001 |
|  | NO FUNCTION | 0.92 | <0.0001 |
| REVEL | RAS-GAP | 0.95 | <0.0001 |
|  | CRAL-TRIO | 0.93 | 0.0002 |
|  | PH LIKE | 0.94 | 0.0075 |
|  | ARMADILLO | 0.9 | <0.0001 |
|  | NO FUNCTION | 0.94 | <0.0001 |
| CLINPRED | RAS-GAP | 0.6 | <0.0001 |
|  | CRAL-TRIO | 0.73 | <0.0001 |
|  | PH LIKE | 0.66 | <0.0001 |
|  | ARMADILLO | 0.71 | <0.0001 |
|  | NO FUNCTION | 0.82 | <0.0001 |
