# Supplementary material for: In-silico Analysis of NF1 Missense Variants in ClinVar: Translating Variant Predictions into Variant Interpretation and Classification
Source: Int J Mol Sci. 2020 Jan 22;21(3):721. doi: 10.3390/ijms21030721 (PMC7037781; doi:10.3390/ijms21030721)
Supplement: Supplementary file 1 [file ijms-21-00721-s001.zip › ijms-614345-supplementary files/Supplementary Table S2.docx]

**Supplementary Table S2**

Cutoff values for pathogenicity identified by different performance indicators

| **Predictor** | **Cutoffs with max Accuracy *(accuracy value)*** | **Cutoffs with max MCC**  ***(MCC value)*** | **Cutoffs with max Youden J-index**  ***(J value)*** | **Cutoffs with max TPR at 10% FPR**  ***(TPR value)*** | **Cutoff with min FPR at 95% TPR *(FPR value)*** |
| --- | --- | --- | --- | --- | --- |
| VEST3 | **0.944** *(0.88)* | **0.944** *(0.69)* | **0.843** *(0.72)* | **0.829** *(0.81)* | **0.691** *(0.33)* |
| REVEL | **0.330** *(0.86)* | **0.330** *(0.65)* | **0.360** *(0.65)* | **0.587** *(0.70)* | **0.224** *(0.47)* |
| ClinPred | **0.583** *(0.89)* | **0.583** *(0.72)* | **0.958** *(0.64)* | **0.987** *(0.71)* | **0.817** (0.65) |

In parenthesis, in italics, are reported the performance indicator values for each cutoff. MCC= Matthews correlation coefficient; TPR= true positive rate; FPR= false positive rate.
